# Non-contrast-enhanced multiparametric cardiac magnetic resonance reveals coronary microvascular functional and structural obstruction after percutaneous coronary intervention

**DOI:** 10.1007/s00330-025-11496-2

**Published:** 2025-03-17

**Authors:** Hideo Arai, Masateru Kawakubo, Pandji Triadyaksa, Adi Wibowo, Kenichi Sanui, Hiroshi Nishimura, Toshiaki Kadokami

**Affiliations:** 1 Fukuokaken Saiseikai Futsukaichi Hospital, Fukuoka, Japan; 2 Department of Health Sciences, Faculty of Medical Sciences, Kyushu University, Fukuoka, Japan; 3 Department of Physics, Faculty of Science and Mathematics, Universitas Diponegoro, Semarang, Indonesia; 4 Department of Informatics, Faculty of Science and Mathematics, Universitas Diponegoro, Semarang, Indonesia

**Keywords:** Coronary microvascular functional and structural obstruction, Acute myocardial infarction, Percutaneous coronary intervention, Multiparametric cardiac magnetic resonance, non-contrast

## Abstract

**Objectives:**

Coronary microvascular functional and structural obstructions (CMVO) after percutaneous coronary intervention (PCI) are a major cause of poor clinical outcomes in patients with acute coronary syndrome. This study aimed to noninvasively diagnose the presence of CMVO using non-contrast multiparametric cardiac magnetic resonance (CMR) in patients with acute myocardial infarction (AMI) who underwent PCI.

**Methods:**

We retrospectively enrolled consecutive patients with AMI who underwent PCI and subsequent acute-phase CMR at our hospital. The patients were divided into two groups: those with and those without CMVO. The top five clinical and CMR parameters were extracted based on their correlation coefficients with the presence of CMVO. Receiver-operator characteristic (ROC) curves and area under the curve (AUC) were generated to compare the diagnostic performance of CMVO detection using the Top_5 clinical parameters, Top_5 CMR parameters, and CMR left ventricular (LV) volume and structure parameters. Differences in the AUC between parameters were compared using the DeLong test.

**Results:**

Forty-eight patients (40 men and 8 women; mean age, 66 ± 12 years) were included in the study. For CMVO detection, the ROC curves of Top_5 clinical parameters, Top_5 CMR parameters, and CMR LV volume and structure parameters demonstrated AUCs of 0.87, 1.00, and 0.72, respectively. The Top_5 CMR parameters exhibited the highest AUC, showing significant differences compared to the other groups.

**Conclusion:**

Non-contrast enhanced multiparametric CMR allows the diagnosis of CMVO with high accuracy and without kidney burden and is expected to be a useful marker for risk stratification, patient management, and treatment decision-making.

**Key Points:**

***Question*** *CMVO following PCI is difficult to diagnose through coronary angiography and can lead to adverse outcomes*.

***Findings*** *Non-contrast enhanced multiparametric CMR imaging has the potential to accurately diagnose CMVOs and further identify their location and extent*.

***Critical relevance*** *Non-contrast enhanced multiparametric CMR enables accurate, noninvasive diagnosis of CMVO, and provides both organic and functional myocardial information. These findings are crucial for diverse CMVO etiologies that require individualized treatment, and may help risk stratification, patient management, and treatment decision-making*.

**Graphical Abstract:**

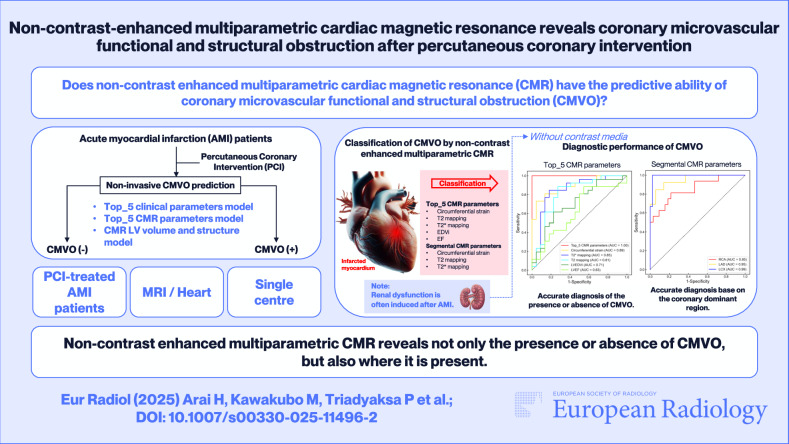

## Introduction

Cardiovascular diseases (CVDs) are the leading cause of death worldwide, with an estimated 17.9 million deaths each year [1]. In recent years, mortality rates have declined due to a decrease in major risk factors for CVDs, such as smoking rates, high cholesterol, and hypertension prevalence, as well as the development of percutaneous coronary intervention (PCI). However, several studies have reported poor prognosis in patients with acute coronary syndrome despite early PCI [2, 3]. One contributing factor is the presence of coronary microvascular functional and structural obstructions (CMVO). CMVO is sometimes expressed as “no-reflow” in coronary angiography (CAG) and is thought to be caused by (i) prolonged ischemia, (ii) distal dispersion of embolic factors, (iii) reperfusion injury, and (iv) individual susceptibility to microvascular dysfunction [4–6]. Notably, CAG, the gold standard for vascular evaluation, does not adequately assess CMVO. Therefore, even with thrombolysis in myocardial infarction (TIMI) grade 3 after PCI, CMVO has been reported in approximately half of these patients [7]. Currently, the simplest and most accurate method to identify CMVO is cardiac magnetic resonance (CMR), namely early gadolinium enhancement (EGE) with contrast media (CM) [8]. It has been reported that CMVO is not only associated with an inability to restore cardiac function but also with an increased likelihood of cardiac death and major adverse cardiovascular events (MACE) [9, 10]. Although CMVO is an important prognostic factor, it is challenging to assess CMVO by EGE with CM because renal dysfunction is often present after acute myocardial infarction (AMI) [11]. Recently, multiparametric CMR, which utilizes various CMR image parameters in clinical practice, has garnered significant attention [12]. We hypothesized that multiparametric CMR analysis without CM could be useful for diagnosing CMVO. This study aimed to noninvasively diagnose CMVO without CM using multiparametric CMR analysis in patients undergoing PCI after AMI.

## Materials and methods

This study was approved by the institutional review board, and the requirement for patient consent was waived. An online provision on the hospital homepage was prospectively made available for patients to opt out of the study. This study was conducted in accordance with the principles of the Declaration of Helsinki.

### Study population

Between August 1, 2018, and May 17, 2020, we retrospectively enrolled consecutive patients with AMI who underwent PCI within 24 h of onset and subsequent acute phase CMR at our hospital. Patients with renal impairment (eGFR < 30) were excluded due to their higher risk of CKD. CMVO was diagnosed as a hypo-intense signal region on EGE images within the infarcted myocardium region on late gadolinium enhancement (LGE) images. The initial diagnosis was made by an analyst with 20 years of clinical experience in cardiac radiology and subsequently confirmed by a senior cardiologist with 35 years of clinical experience, including 20 years specializing in CMR interpretation. To avoid the influence of CMR analysis values, CMVO was first diagnosed with LGE and EGE. According to the American Heart Association (AHA) 16-segment model, a side-by-side visual comparison between the infarcted LGE area and EGE was performed to localize the CMVO area in the corresponding EGE images. At the first visit during the acute phase, the following 32 clinical parameters were recorded: age, sex, height, weight, body mass index, body surface area, heart rate, systolic and diastolic blood pressure, history of arterial fibrillation, stroke, diabetes, hypertension, dyslipidemia, and smoking, values of creatine kinase (CK), hemoglobin (Hb), platelets count (Plt), HbA1c, low-density lipoprotein cholesterol (LDL-C), and Creatinine, antiplatelet, anticoagulant, beta blocker, angiotensin-converting enzyme inhibitors, mineralocorticoid receptor antagonists, angiotensin II receptor blockers, calcium antagonist, onset to balloon time (OTB), door to balloon time (DTB), and pre-TIMI and post TIMI grade.

### CMR protocol

All patients underwent CMR with a 3.0-T MRI system (Ingenia, Philips Healthcare) with a 40-mT/m maximum gradient strength, 200-T/ms slew rate, and a 32-channel phased-array receiver coil. All CMR images were obtained using an ECG-gated and a breath-hold. Cine imaging based on steady-state free precession was acquired for the entire left ventricular (LV) along the short-axis (SA), with 20 reconstructed phases per heartbeat. The cine parameters were as follows: TR/TE, 3.0 ms/1.49 ms; flip angle (FA), 50°; slice thickness, 8 mm; field of view (FOV), 350 × 350 mm^2^; in-plain resolution, 0.99 × 0.99 mm^2^; acquisition matrix, 176 × 201; and sensitivity encoding (SENSE) factor, 2.5. T2 mapping based on gradient and spin-echo was used to acquire three slices of the base, middle, and apex in the SA. The T2 mapping parameters were as follows: TR/first TE/delta TE, cardiac cycle/20 ms/10 ms; number of echoes, 5; FA, 90°; slice thickness, 10 mm; FOV, 350 × 350 mm^2^; in-plain resolution, 0.99 × 0.99 mm^2^; acquisition matrix, 176 × 173; and SENSE factor, 2. T2* mapping based on a gradient echo was used to acquire three slices for T2 mapping. The T2* mapping parameters were as follows: TR/first TE/delta TE, 11 ms/2 ms/2 ms; number of echoes, 5; FA, 25°; slice thickness, 10 mm; FOV, 350 × 350 mm^2^; in-plain resolution, 0.99 × 0.99 mm^2^; acquisition matrix, 176 × 176; and SENSE factor, 2. Cine, T2 mapping, and T2* mapping were performed before CM administration. EGE was performed using 3-dimensional (3D) T1-weighted (T1W) acquired across the entire LV approximately 1–3 min after administering the CM of gadolinium-DO3A-butriol (Gadovist, Bayer; 0.1 mL/kg). LGE imaging based on 3DT1W was performed throughout the entire LV in the SA approximately 10 min after administering the CM. The EGE and LGE parameters were as follows: TR/TE/, 3.2 ms/1.5 ms; FA, 15°; inversion delay time at EGE, approximately 750 ms (longest setting, heart rate dependent); slice thickness, 10 mm; FOV, 350 × 350 mm^2^; in-plain resolution, 0.68 × 0.68 mm^2^; acquisition matrix, 236 × 172; and SENSE factor, 3.2.

### CMR image analysis

Based on the short-axis cine imaging, the left ventricle end-diastolic volume index (LVEDV_i_), left ventricle end-systolic volume index (LVESV_i_), left ventricle ejection fraction (LVEF), and mass index (mass_i_) were manually measured using the Philips Extended MR WorkSpace (version 2.6.3.4; Philips Medical Systems).

Circumferential strain (CS) on the base, midventricular, and apical slices of short-axis images was measured using the off-line feature-tracking software, which has been used and validated in several previous studies [13]. First, the endocardial regions of the LV on the SA cine images were carefully contoured using MATLAB software (MathWorks, Inc.). Subsequently, the motion of the endocardial region was automatically tracked for a cardiac cycle using a template-matching algorithm. Segmental CSs based on the AHA 16-segments were calculated based on the measured CS values [14]. The global CS was defined as the mean of the CSs among the 16 segments.

The T2 values of AHA 16-segments were obtained using the dedicated commercial software SYNAPSE VINCENT (Fujifilm Co.). For the myocardial segmentation according to the AHA model, the LV myocardium was manually traced based on the epicardium and endocardium to determine the region of interest (ROI), carefully excluding all regions that did not represent the myocardium. Then the 16-segments’ T2 values were automatically obtained based on the ROI of the myocardium. The global T2 value was defined as the mean among the 16 segments.

According to the previous method, T2* values were evaluated using MATLAB software (MathWorks, Inc., US) using the least-squares fitting monoexponential method [15]. At the base, mid-ventricular, and apical short-axis locations, pixel-wise T2* analysis was performed to calculate the median T2* value for each segment according to the AHA 16-segment model. The global T2* value was defined similarly to that in the T2 analysis.

### Statistical analysis

The Shapiro–Wilk test was employed to assess the normality of the data distribution. Descriptive statistics were provided as the mean ± standard deviation unless defined otherwise or as the median and interquartile range (IQR) based on normality. Categorized datasets were subjected to significant difference testing using the Student’s *t*-test or Mann–Whitney *U*–test based on normality and chi-square tests for binary variables. Outliers in the CS, T2, and T2* values were processed using the IQR method and replaced with the mean to reduce the effect of outliers.

The patients were categorized into two groups: those with and those without CMVO. Global values were calculated as the average of CS, T2, and T2*. The clinical parameters, CMR LV volume and structure parameters, and the global values of CS, T2, and T2* were analyzed. Additionally, their correlation with the presence of CMVO was analyzed using the correlation coefficients. According to the correlation coefficients, the top five parameters were chosen as the clinical and CMR parameters. CMR LV volume and structure parameters were included LVEDVi, LVESVi, LVEF, and massi. The contribution of each parameter to the predictive value of the model was calculated by odds ratio in univariate logistic regression analysis. Additionally, the impact of parameter interdependence on the model’s predictive value was evaluated through odds ratios obtained from multivariate logistic regression analysis. All continuous variables underwent *z*-score normalization. Receiver-operator characteristics (ROC) curves and area under curve (AUC) were created to compare the diagnostic performance of the Top_5 clinical parameters, Top_5 CMR parameters, and CMR LV volume and structure parameters to detect CMVO. The optimal cut-off values, sensitivity, and specificity for the ROCs were calculated with the Youden Index [16]. The AUC’s 95% confidence intervals (CI) were calculated using bootstrap CI. Differences in AUCs between the parameter groups were compared using the DeLong test.

The values of CS, T2, and T2* across the AHA 16 segments in all patients were categorized into groups with and without CMVO. The 16-segment values were divided into three parts according to the dominant region: the left anterior descending artery (LAD: segments #1, #2, #7, #8, #13, and #14), the right coronary artery (RCA: segments #3, #4, #9, #10, and #15), and the left circumflex artery (LCX: segments #5, #6, #11, #12, and #16). All continuous variables underwent *z*-score normalization. Logistic regression was used to combine the parameters of the three groups. ROC analyses were performed to evaluate the predictive ability of CMVO presence in a manner similar to that of global values.

Intra- and inter-observer agreement of the measurements was assessed using the intraclass correlation coefficient (ICC). Intra-observer agreement was calculated using ICC (1, 1), based on a one-way random-effects model, while inter-observer agreement was determined using ICC (2, 1), based on a two-way random-effects model. Measurements were repeated at least one week apart in 15 randomly selected patients by both the primary analyst and an additional analyst, each with over 20 years of clinical experience in cardiac radiology. ICC values were classified as excellent (ICC ≥ 0.90), good (ICC = 0.75–0.89), moderate (ICC = 0.50–0.74), and poor (ICC < 0.50), in accordance with previous literature [17].

Statistical significance was set at *p* = 0.05. All statistical analyses were performed using the stats module in Python 3.9.13 and the ‘pROC’, ‘statsmodels’, and ‘irr’ packages in R version 4.4.0 [18, 19].

## Results

### Study population

**Fig. 1:**
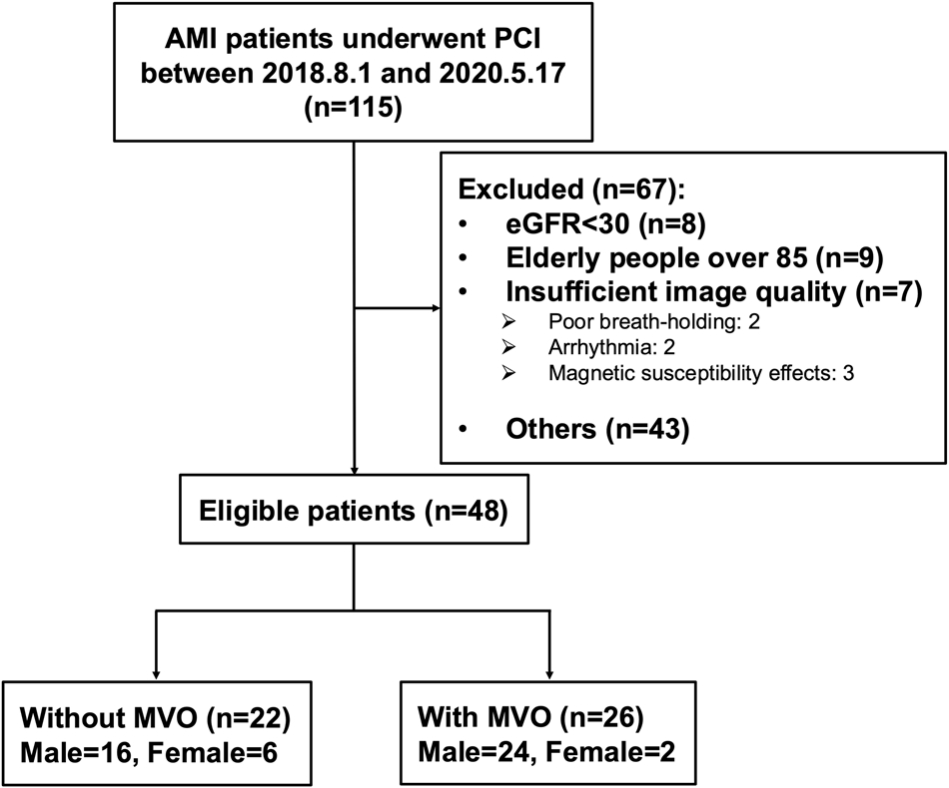
Flowchart of patient selection in this study

**Fig. 2:**
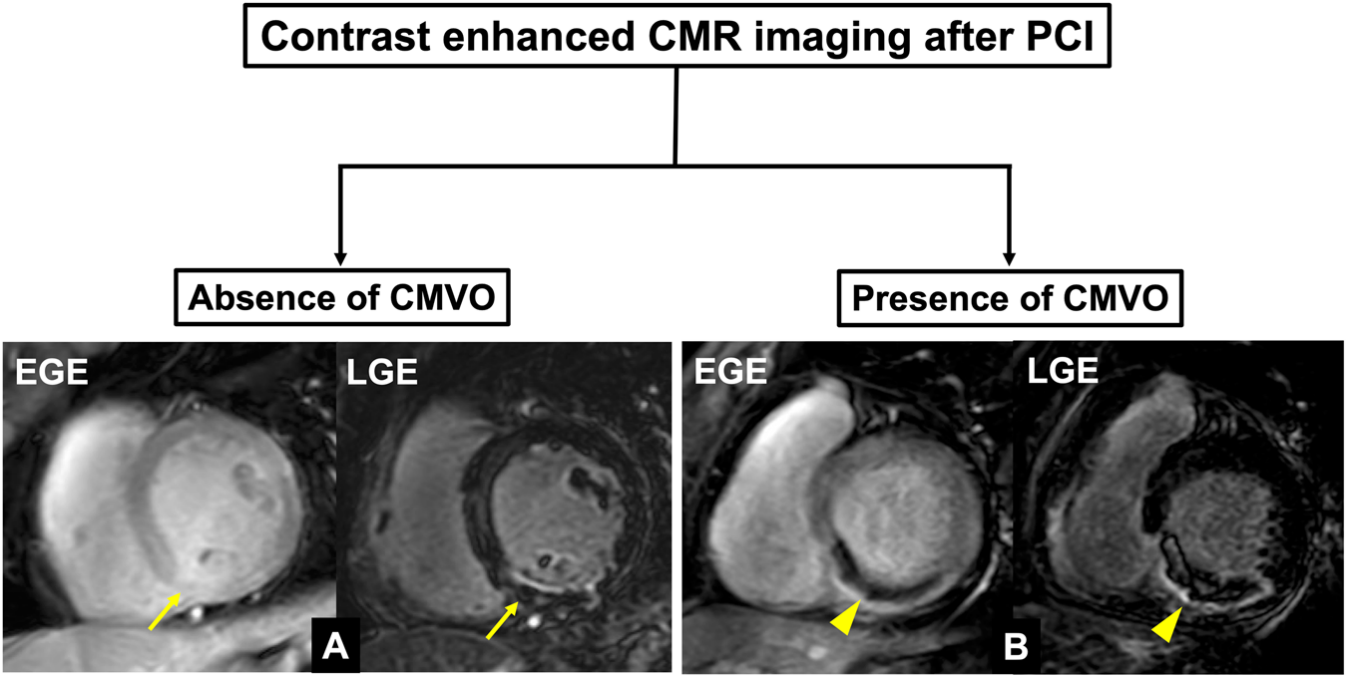
Flowchart for diagnosing the presence or absence of CMVO using contrast-enhanced EGE and LGE imaging of CMR. **A** shows the absence of CMVO, and **B** shows the presence of CMVO. On the intimal side of the infarcted myocardium region diagnosed by the LGE, a hyper-intense myocardial signal on the EGE indicates the absence of CMVO (the yellow arrow on the EGE [**A**]), while a hypo-intense myocardial signal on the EGE indicates the presence of CMVO (the yellow arrowhead on the LGE [**B**])

**Fig. 3:**
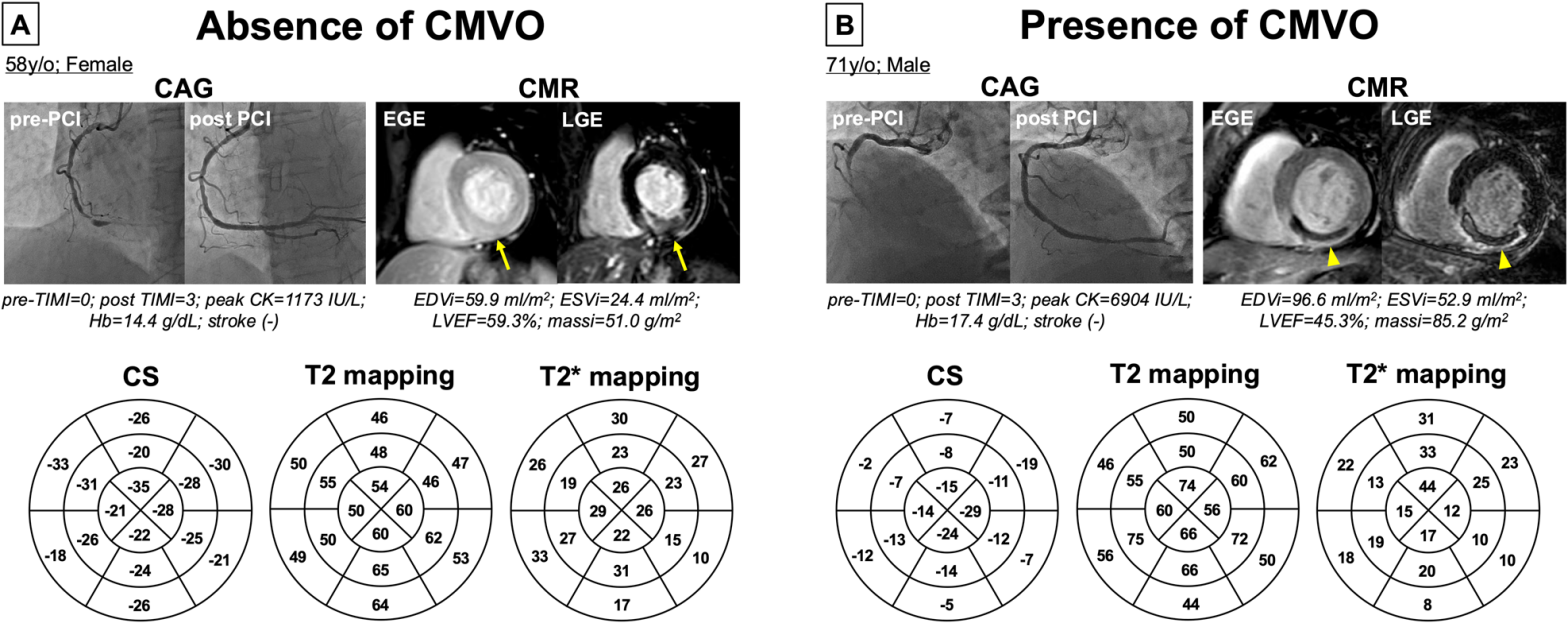
**A**, **B** Show CAG and CMR images and analysis results of AMI patients who underwent PCI in the right coronary artery (RCA). **A** Case without CMVO; LGE depicted high signal intensity mixed with some pale areas indicating inflammation and fibrosis. EGE did not show low signal intensity indicative of CMVO. Increased edema was observed due to elevated T2 mapping, but no wall motion reduction due to reduced CS and no intramyocardial hemorrhage due to decreased T2 and T2* mapping. **B** Case with CMVO; in the RCA region, LGE depicted low signal intensity indicating CMVO amidst high signal intensity indicative of inflammation and fibrosis. Additionally, EGE showed low signal intensity indicative of CMVO. In this region, wall motion reduction was observed due to decreased CS, along with increased edema from elevated T2 mapping and intramyocardial hemorrhage due to decreased T2 and T2* mapping

This study included 115 patients who underwent PCI after AMI. Sixty patients did not undergo CMR and were therefore excluded from this study (eGFR < 30 in 8, elderly people over 85 in 9, and others in 43 patients). After excluding 7 patients due to insufficient image quality (poor breath-holding: 2; arrhythmia: 2; magnetic susceptibility effects: 3), 48 patients (66 ± 12 years, 83% male) were included in the present analysis (Fig. 1). Figure 2 depicts the representative image for the diagnosing the presence or absence of CMVO using contrast-enhanced EGE and LGE CMR imaging. LGE was identified in 46 (96%) patients, with 26 (54%) patients additionally experiencing CMVO. The patient characteristics are presented in Table 1. The normal reference ranges for CS, T2, and T2* values at our hospital were -21.3 ± 5.3%, 47.0 ± 3.8 ms, and 24.2 ± 9.4 ms, respectively. Figure 3 shows representative cases with and without CMVO using CAG and CMR images, and corresponding analysis results.

**Table 1:** Patient characteristics

	Total *n* = 48	Without CMVO *n* = 22	With CMVO *n* = 26	*p*-value
Clinical data				
STEMI, *n* (%)	42 (88)	18 (82)	24 (92)	0.27
Age, y	66 ± 12	67.7 ± 9.8	64.4 ± 13.9	0.35
Male, *n* (%)	40 (83)	16 (73)	24 (92)	0.07
Body mass index, kg/m^2^	24.8 ± 2.7	24.9 ± 2.6	24.7 ± 2.9	0.89
Heart rate, bpm	63 ± 10	64 ± 10	62 ± 9	0.44
Systolic BP, mmHg	133 ± 31	139 ± 27	128 ± 35	0.23
Diastolic BP, mmHg	83 ± 27	82 ± 15	83 ± 27	0.94
Atrial fibrillation, *n* (%)	3 (6)	2 (9)	1 (4)	0.45
Hypertension, *n* (%)	26 (54)	12 (55)	14 (54)	0.96
Diabetes mellitus, *n* (%)	19 (40)	9 (41)	10 (38)	0.86
Hyperlipidemia, *n* (%)	27 (56)	12 (55)	15 (58)	0.83
Stroke, *n* (%)	3 (6)	0 (0)	3 (12)	0.10
Current smoker, *n* (%)	22 (46)	11 (50)	11 (50)	0.59
Biochemical data				
Peak CK, IU/L	1263 [552–4957]	605 [367–1142]	4153 [1159–6298]	< 0.01^**^
Hb, g/dL	14.5 ± 1.6	14.7 ± 1.3	14.3 ± 1.8	0.36
Plt, /μL	25.3 [20.7–216.8]	28.2 [21.2–193.3]	25.2 [20.8–224.3]	0.77
HbA1c, %	6.3 [6.0–7.2]	6.1 [5.9–7.2]	6.4 [6.0–7.2]	0.47
LDL-C, mg/dL	111 [97–142]	106 [96–151]	123 [101–142]	0.58
Cre, mg/dL	0.92 [0.74–1.07]	0.89 [0.73–1.01]	0.94 [0.76–1.12]	0.32
Angiographic data				
Culprit lesion				0.79
RCA, *n* (%)	19 (40)	8 (36)	11 (42)	
LAD, *n* (%)	26 (54)	13 (59)	13 (50)	
LCX, *n* (%)	3 (6)	1 (5)	2 (8)	
Number of lesions				0.75
1-vessel disease, *n* (%)	31 (65)	14 (64)	17 (65)	
2-vessel disease, *n* (%)	9 (19)	5 (23)	4 (17)	
3-vessel disease, *n* (%)	8 (17)	3 (14)	5 (19)	
OTB, min	284 [147–627]	305 [141–816]	284 [153–575]	0.79
DTB, min	66 [53–83]	66 [54–79]	66 [53–87]	0.91
TIMI flow grade				
0/1 Pre-PCI, *n* (%)	39 (81)	15 (68)	24 (92)	0.03^*^
3 Post PCI, *n* (%)	45 (94)	21 (95)	24 (92)	0.65
CMR data				
LVEDVi, mL/m^2^	80.0 ± 17.3	73.5 ± 16.1	85.5 ± 16.5	0.01^*^
LVESVi, mL/m^2^	41.7 ± 15.8	36.6 ± 13.4	46.0 ± 16.6	0.04^*^
LVEF, %	49.3 ± 9.9	51.3 ± 9.3	47.6 ± 10.3	0.19
LV mass index, g	63.0 ± 11.9	63.0 ± 12.1	63.0 ± 11.9	0.99
Global CS, %	−16.8 [−21.5–12.5]	−17.4 [−22.7–12.9]	−14.9 [−19.6–10.7]	< 0.01^**^
Global T2 value, ms	55 [48–62]	53 (46–60)	55 [47–62]	0.07
Global T2* value, ms	22.1 [17.0–26.2]	22.1 [16.5–26.4]	21.3 [15.6–25.6]	0.12
LGE, *n* (%)	46 (96)	20 (91)	26 (100)	0.12

Data as mean ± SD, median [IQR], or *n* (%)

*CMVO* coronary microvascular functional and structural obstruction, *STEMI* ST-elevation myocardial infarction, *BP* blood pressure, *RCA* right coronary artery, *LAD* left anterior descending artery, *LCX* left circumflex artery, *OTB* onset to balloon time, *DTB* door to balloon time, *TIMI* thrombolysis in myocardial infarction, *PCI* percutaneous coronary intervention, *CMR* cardiac magnetic resonance, *LVEDVi* left ventricle end-diastolic volume index, *LVESVi* left ventricle end-systolic volume index, *LVEF* left ventricle ejection fraction, *CS* circumferential strain, *LGE* late gadolinium enhancement

^*^ Significant *p*-value < 0.05

^**^ Most significant *p*-value < 0.01

### Selection Top_5 clinical and CMR parameters

**Fig. 4:**
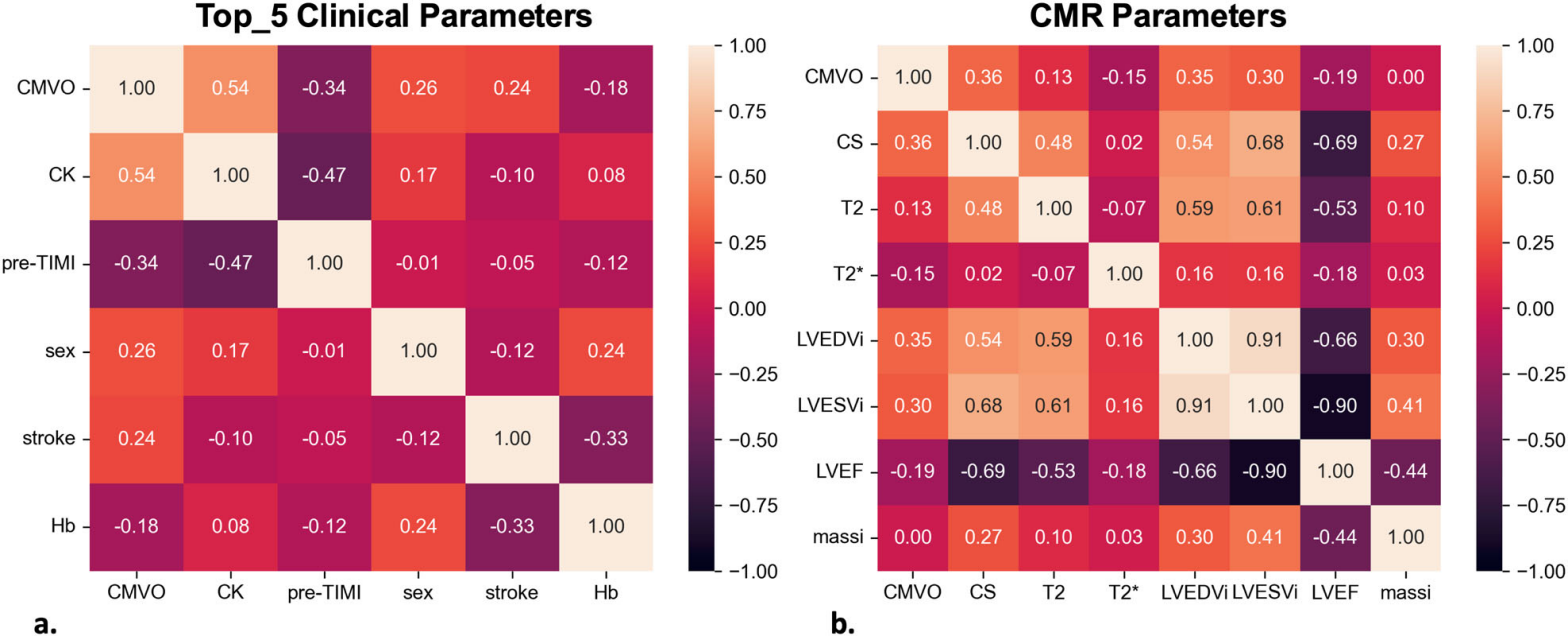
Heatmaps on the correlation coefficients of **a** Top_5 clinical parameters and **b** CMR parameters with and without CMVO

The correlation coefficients of the clinical and CMR parameters with respect to CMVO were analyzed using Spearman’s correlation and Pearson’s correlation, respectively (Fig. 4). In the correlation analysis, the Top_5 clinical parameters selected were CK, pre-TIMI, sex, stroke, and Hb. The Top_5 CMR parameters selected were T2 mapping, T2* mapping, CS, LVEDVi, and LVEF. LVESV_i_ had the fifth highest correlation; however, it was excluded because of its high correlation with LVEDV_i_.

### Diagnostic performance

**Fig. 5:**
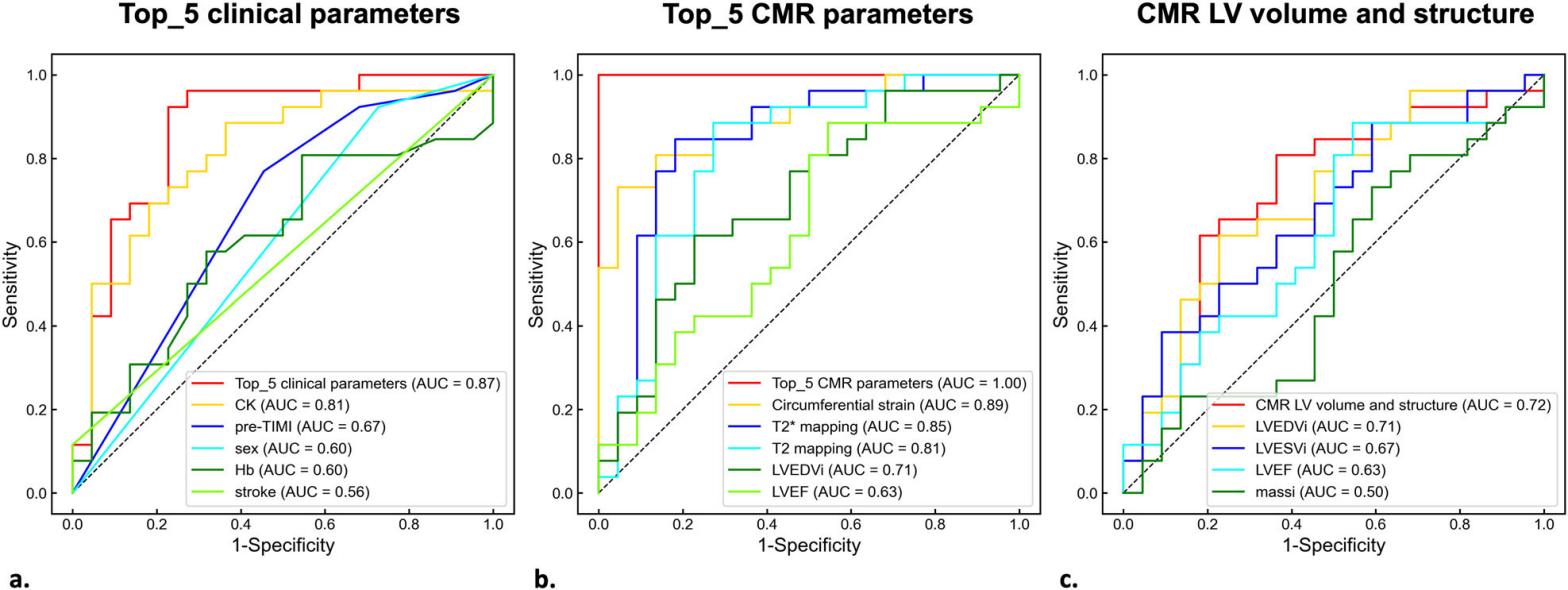
ROC curves for detecting CMVO after PCI using three-parameter groups. The legends of Top_5 clinical parameters (**a**), Top_5 CMR parameters (**b**), and CMR LV volume and structure parameters (**c**) are composed of the combined parameters and each of the parameters that comprise them. The AUC of the Top_5 CMR parameters is statistically significantly higher than that of the other parameter groups (DeLong test, Top_5 CMR parameters vs Top_5 clinical parameters: *p* = 0.02; Top_5 CMR parameters vs CMR LV parameters: *p* < 0.01; CMR LV parameters vs Top_5 clinical parameters: *p* = 0.05)

For CMVO detection, ROC curves of Top_5 clinical parameters, Top_5 CMR parameters, and CMR LV volume and structure parameters demonstrated AUCs of 0.87 [95% CI: 0.74–0.96], 1.00 [95% CI: 1.00–1.00], and 0.72 [95% CI: 56–86], respectively (Fig. 5). A comparison of the AUCs among the three groups using the DeLong test revealed that the Top_5 CMR parameter with the highest AUC exhibited significant differences compared to the other groups (Top_5 CMR vs Top_5 clinical: *p* = 0.02; Top_5 CMR vs CMR LV: *p* < 0.01; CMR LV vs Top_5 clinical: *p* = 0.05). Table 2 presents the diagnostic performance of individual numerical parameters optimized for sensitivity and specificity in identifying CMVO.

**Table 2:** Identification of CMVO with parameters optimized for sensitivity and specificity

Variables	Cutoff value	Sensitivity (%)	Specificity (%)	AUC	95% CI
Top_5 clinical parameters		92	77	0.87	0.74–0.96
CK, IU/L	4124	88	64	0.81	0.67–0.92
Pre-TIMI	1.2	77	55	0.67	0.54–0.80
Sex, male		92	27	0.60	0.50–0.71
Hb, g/dL	15.3	81	45	0.60	0.44–0.77
Stroke		12	100	0.56	0.50–0.63
Top_5 CMR parameters		100	100	1.00	1.00–1.00
CS, %	−12.8	73	95	0.89	0.78–0.96
T2* value, ms	25	85	82	0.85	0.73–0.95
T2 value, ms	61	88	73	0.81	0.66–0.92
LVEDVi, mL/m^2^	90.3	62	77	0.71	0.55–0.84
LVEF, %	54.0	88	45	0.63	0.44–0.79
CMR LV volume and structure		81	64	0.72	0.56–0.86
LVEDVi, mL/m^2^	90.3	62	77	0.71	0.55–0.84
LVESVi, mL/m^2^	51.9	38	91	0.67	0.51–0.82
LVEF, %	54.0	88	45	0.63	0.44–0.79
Mass index, g/m^2^	69.3	73	41	0.50	0.33–0.67

*CMVO* coronary microvascular functional and structural obstruction, *AUC* area under the curve, *CI* confidence interval, *CK* creatine kinase, *Hb* hemoglobin, *CMR* cardiac magnetic resonance, *CS* circumferential strain, *LV* left ventricle, *LVEDVi* left ventricle end-diastolic volume index, *LVESVi* left ventricle end-systolic volume index, *LVEF* left ventricle ejection fraction

**Fig. 6:**
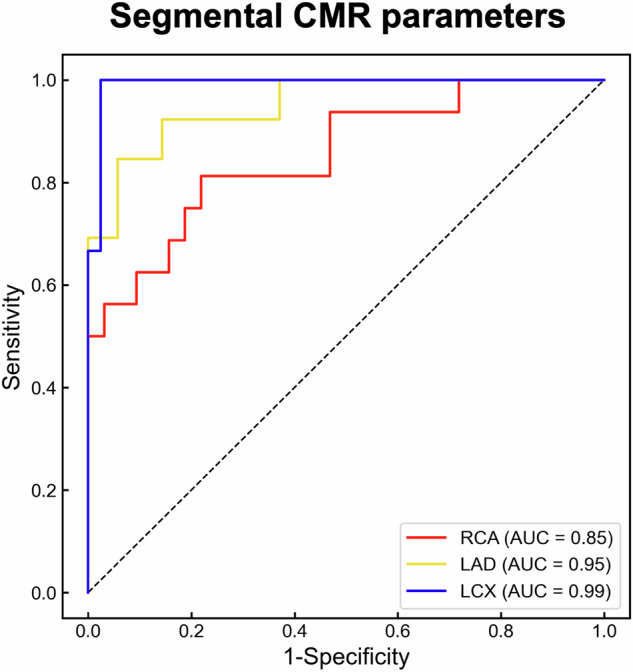
ROC curves for detecting regional CMVO after PCI using segmental CMR mapping consist of T2 mapping, T2* mapping, and CS. Segmental CMR mapping allows for the detection of the presence or absence of regional CMVO with high accuracy based on the coronary dominant region (AUC_RCA_ 0.85, 95% CI_RCA_: 0.72–0.96; AUC_LAD_ 0.95, 95% CI_LAD_: 0.88–1.00; AUC_LCX_ 0.99, 95% CI_LCX_: 0.97–1.00)

Segmental CMR parameters consisting of T2 mapping, T2* mapping, and CS demonstrated high AUCs of 0.85 [95% CI: 0.72–0.96], 0.95 [95% CI: 0.88–1.00], and 0.99 [95% CI: 0.97–1.00] for RCA, LAD, and LCX, respectively (Fig. 6).

### Contribution of parameters

In the univariate logistic regression analysis, the odds ratios for the clinical parameters CK and Pre-TIMI were significantly different, at 3.71 and 0.46, respectively. For the CMR parameters, the odds ratios for mean CS, LVEDVi, and LVESVi were significantly different, at 2.22, 2.18, and 1.95, respectively (Table 3). In the multivariate logistic regression analysis, the odds ratios for CK and sex within the Top_5 clinical parameters were significantly different, at 4.94 and 3.44, respectively. For the Top_5 CMR parameters, the odds ratio for LVEDVi was significantly different, at 3.31 (Table 4).

**Table 3:** Odds ratio in univariate logistic regression

	Odds ratio (95% CI)	*p*-value
Clinical data		
CK	3.71 (1.46, 9.44)	< 0.01^**^
Hb	0.76 (0.42, 1.36)	0.35
Plt	0.93 (0.53, 1.65)	0.81
HbA1c	1.32 (0.71, 2.42)	0.38
LDL_C	0.97 (0.55, 1.71)	0.91
Cre	1.44 (0.75, 2.76)	0.27
Antiplatelet	2.00 (0.51, 7.84)	0.32
Anticoagulant	1.80 (0.50, 6.50)	0.37
Beta-blocker	2.74 (0.26, 28.41)	0.40
ACEI	–	–
MRA	–	–
ARB	0.80 (0.22, 2.96)	0.74
Calcium antagonist	0.63 (0.16, 2.46)	0.51
Years	0.75 (0.42, 1.35)	0.34
Sex	1.75 (0.92, 3.33)	0.09
Height	1.22 (0.69, 2.16)	0.50
Weight	1.09 (0.62, 1.93)	0.76
BMI	0.96 (0.54, 1.69)	0.89
HR	0.80 (0.45, 1.42)	0.44
Systolic BP	0.70 (0.39, 1.25)	0.23
Diastolic BP	1.02 (0.58, 1.81)	0.94
AF	0.40 (0.03, 4.74)	0.47
Stroke	–	–
Diabetes	0.90 (0.28, 2.88)	0.86
Hypertension	0.97 (0.31, 3.04)	0.96
Dyslipidemia	1.14 (0.36, 3.57)	0.83
Smoking	0.73 (0.23, 2.30)	0.59
BSA	1.14 (0.64, 2.01)	0.66
DTB	0.94 (0.53, 1.65)	0.82
OTB	0.69 (0.31, 1.54)	0.36
Pre-TIMI	0.46 (0.22, 0.94)	0.03^*^
Post-TIMI	0.87 (0.22, 3.44)	0.84
CMR data		
Mean T2	1.30 (0.73, 2.32)	0.38
Mean T2^*^	0.73 (0.41, 1.31)	0.29
CS	2.22 (1.13, 4.35)	0.02^*^
LVEDVi	2.18 (1.13, 4.24)	0.02^*^
LVESVi	1.95 (1.01, 3.76)	0.045^*^
LVEF	0.67 (0.37, 1.22)	0.19
massi	1.00 (0.57, 1.77)	0.99

*CI* confidence interval, *CMR* cardiac magnetic resonance, *CK* creatine kinase, *CS* circumferential strain, *LVEDVi* left ventricle end-diastolic volume index, *LVESVi*, *ACEI* angiotensin-converting enzyme inhibitor, *Cre* creatinine, *BSA* body surface area, *BP* blood pressure, *massi* mass index, *MRA* mineralocorticoid receptor antagonists, *LDL-C* low-density lipoprotein cholesterol, *BMI* body mass index, *DTB* door to balloon time, *Plt* platelets count, *TIMI* thrombolysis in myocardial infarction, *ARB* angiotensin II receptor blockers, *HR* heart rate, *Hb* hemoglobin, *OTB* onset to balloon time, *LVEF* left ventricle ejection fraction

^*^ Significant *p*-value < 0.05

^**^ Most significant *p*-value < 0.01

“–“ Indicates that the data could not be measured or analyzed due to variability in the data or small sample sizes

**Table 4:** Odds ratio in multivariate logistic regression

	Odds ratio (95% CI)	*p*-value
Top_5 clinical parameters		
CK	4.94 (1.35, 18.12)	0.02^*^
Pre-TIMI	0.50 (0.20, 1.27)	0.15
Sex	3.44 (1.15, 10.33)	0.03^*^
Hb	0.42 (0.16, 1.10)	0.08
stroke	–	–
Top_5 CMR parameters		
Mean T2	0.56 (0.23, 1.39)	0.21
Mean T2*	0.54 (0.26, 1.15)	0.13
Mean CS	2.73 (1.00, 7.47)	0.05
LVEDVi	3.31 (1.17, 9.31)	0.02^*^
LVEF	1.64 (0.59, 4.53)	0.34
CMR LV volume and structure		
LVEDVi	1.11 (0.02, 67.97)	0.96
LVESVi	4.07 (< 0.01, > 10^3^)	0.71
LVEF	2.19 (0.04, > 10^2^)	0.69
massi	0.78 (0.40, 1.55)	0.49

*CI* confidence interval, *CK* creatine kinase, *TIMI* thrombolysis in myocardial infarction, *Hb* hemoglobin, *CMR* cardiac magnetic resonance, *CS* circumferential strain, *LVEDVi* left ventricle end-diastolic volume index, *LV* left ventricle, *LVESVi* left ventricle end-systolic volume index, *LVEF* left ventricle ejection fraction, *massi* mass index

^*^ Significant *p*-value < 0.05

“–“ Indicates that the data could not be measured or analyzed due to variability in the data or small sample size

### Intra- and inter-observer agreement

The ICCs (1,1) for segmental CS, T2, and T2* were 0.65 (95% CI: 0.51–0.75), 0.99 (95% CI: 0.98–0.99), and 0.95 (95% CI: 0.93–0.97), respectively, and ICC (1, 1) of CS indicated moderate agreement, ICC (1, 1) of T2 and T2* indicated excellent agreement (Table 5). Similarly, ICCs (2, 1) for segmental CS, T2, and T2* were 0.57 (95% CI: 0.41–0.70), 0.92 (95% CI: 0.89–0.95), and 0.91 (95% CI: 0.87–0.94), respectively, and ICC (2, 1) of CS indicated moderate agreement, ICC (2, 1) of T2 and T2* indicated excellent agreement.

**Table 5:** Intra- and inter-observer agreement: ICCs of CS, T2 mapping, and T2* mapping with 95% CIs

	ICC (95% CI)
Intra-observer agreement	
Global CS	0.83 (0.57, 0.94)
Global T2 mapping	0.995 (0.986, 0.998)
Global T2* mapping	0.95 (0.87, 0.98)
Segmental CS	0.65 (0.51, 0.75)
Segmental T2 mapping	0.99 (0.98, 0.99)
Segmental T2* mapping	0.95 (0.93, 0.97)
Inter-observer agreement	
Global CS	0.68 (0.25, 0.88)
Global T2 mapping	0.98 (0.94, 0.99)
Global T2* mapping	0.92 (0.79, 0.97)
Segmental CS	0.57 (0.41, 0.70)
Segmental T2 mapping	0.92 (0.89, 0.95)
Segmental T2* mapping	0.91 (0.87, 0.94)

*ICC* confidence interval, *CS* circumferential strain, *CI* confidence interval

## Discussion

In this study, we assessed the detectability of CMVO in patients with AMI who underwent PCI using three sets of parameters comprised of clinical and CMR information. After AMI, patients often experience impaired renal function; however, our proposed methods are highly versatile owing to the absence of CM. We found that the Top_5 CMR parameters could detect CMVO with the highest accuracy.

### Feature selection

Correlation coefficients were employed to identify parameters, with those demonstrating strong linear relationships being selected. Univariate and multivariate logistic regression analyses were then performed to evaluate nonlinear associations, causal relationships, and interaction effects among the parameters. In the odds ratios from univariate logistic regression, the parameters that exhibited significant differences included CK and Pre-TIMI for the clinical parameters and CS, LVEDVi, and LVESVi for the CMR parameters. These parameters corresponded with the top five parameters derived from the correlation coefficients, validating that the feature selection for the model based on these coefficients was appropriate. In the odds ratios obtained from multivariate logistic regression, significantly different parameters among the Top_5 clinical parameters were CK and sex and LVEDVi among the Top_5 CMR parameters. These parameters were shown to contribute significantly and independently to improving the accuracy of CMVO detection, even within the context of interdependence among the parameters.

### CMVO diagnosis

Factors associated with CMVO vary and include prolonged ischemia, distal dispersion of embolic factors, reperfusion injury, and individual susceptibility to microvascular dysfunction [4–6]. The Top_5 clinical parameters extracted from this clinical information can be used to detect CMVO with high accuracy. Among these clinical parameters, CK exhibited the highest AUC, contributing to the improved accuracy of detection. Myocardial markers, such as CK, are used not only as detection markers for AMI but also as indicators of infarct size [20]. Because an increased infarct area is a risk factor for CMVO, it is understandable that CK demonstrated a strong correlation with CMVO. The pre-TIMI demonstrated the second-highest AUC for detecting CMVO. This can be explained by the degree of ischemia associated with CMVO [21]. However, the AUCs for the other three parameters were less than 0.6, which may not have sufficiently contributed to improving the accuracy of CMVO detection.

The Top_5 CMR parameters had the highest AUC for detecting CMVO. Among the Top_5 CMR parameters, CS had the highest AUC, contributing to the improved accuracy. A previously reported strain analysis revealed high detectability of CMVO (AUC 0.764), consistent with our findings [22]. The presence of CMVO has been reported to correlate with myocardial infarct size and severity of myocardial damage [5]. Additionally, considering the orientation of myocardial fibers, the endocardial layer primarily contributes to longitudinal contraction, while the mid-myocardial layer is primarily involved in circumferential contraction. Since myocardial damage due to ischemia typically occurs from the endocardial side, more severe myocardial damage can also affect circumferential contraction [23]. This suggests that CMVO is more likely to occur in cases of more severe myocardial injury with reduced CS. In this study, CS demonstrated a significant difference, with an odds ratio of 2.22 observed in univariate regression analysis. In multivariate regression, the odds ratio (95% CI) was 2.73 (1.00–7.47) at *p* = 0.05; however, this result, while noteworthy, did not reach statistical significance. One potential explanation for this finding is the moderate ICC of segmental CS. The reproducibility of CS in this study is considered reasonable, given that segmental strain is generally recognized to be less reproducible than global strain [24, 25]. Despite these limitations, the AUC of CS indicated strong CMVO detection capability. These findings suggest that regional wall motion abnormalities can be effectively detected, even though the reproducibility of CS is limited. Furthermore, the robustness of these results reinforces the potential utility of CS in this context. Nevertheless, future technological advancements aimed at improving the reproducibility of CS are anticipated to further enhance its utility in CMVO detection. Additionally, the other segmental CMR parameters of T2 and T2* mappings showed high AUCs. Segmental CMR parameters provide regional information on myocardial characteristics; CS is regional motion, T2 mapping is regional edema, and T2* mapping is regional intramyocardial hemorrhage [26–28]. This segmental information can provide local myocardial characteristics that cannot be acquired using the Top_5 clinical parameters. We believe that information regarding the type and extent of regional myocardial damage contributes to the improved detectability of CMVO. Regarding LVEF and LVEDVi, key global indicators among the Top 5 CMR parameters, it is recognized that following severe myocardial infarction, LVEF decreases while LVEDVi expands to maintain cardiac output [29]. In this way, the Top_5 CMR parameters reflect not only multifaceted information regarding myocardial infarction but also local and global information, which we believe contributed to the improved accuracy. In contrast, CMR LV volume and structure parameters did not provide sufficient CMVO detection ability. Because AMI is a localized disease in the myocardium, there may be limitations to using CMR LV volume and structure parameters when evaluating the entire heart. It has been reported that peak strain, as a local indicator, is superior to LVEF, which is an overall indicator, in detecting myocardial infarction [30].

In the current study, the segmental CMR parameters, CS, T2 mapping, and T2* mapping played major roles in detecting CMVO. Although the Top_5 clinical parameters had high CMVO detection performance, they did not provide regional information. In contrast, regional information on edema, bleeding, and motion obtained from segmental CMR parameters is valuable for both detecting CMVO and managing patients, including preventing complications, by providing a more accurate picture of myocardial injury severity [31]. Notably, the segmental CMR parameters enabled regional CMVO detection with high accuracy (AUC_RCA_ 0.85; AUC_LAD_ 0.95; and AUC_LCX_ 0.99). However, this study lacked an external validation group due to its small size, which may lead to concerns regarding outfitting and generalization performance. Although the AUC for the top 5 CMR parameters showed 1.0, further evaluation of performance across different contexts is necessary.

The development of CMVO promotes remodeling, increases cardiovascular events, and worsens the prognosis [10]. Information regarding the presence or absence of CMVO cannot be obtained from clinical data or standard CAG. The gold standard for CMVO diagnosis involves coronary perfusion velocimetry using a Doppler wire during CAG, but this technique requires specialized equipment, and additional pharmacological interventions, and is more invasive [4]. In contrast, the gold standard for noninvasive CMVO detection is contrast-enhanced CMR, and our proposed Top_5 CMR parameters enable highly accurate CMVO detection without the need for CM. Furthermore, segmental CMR mapping allowed for regional CMVO detection. Based on these findings, multiparametric CMR analysis may be a useful prognostic indicator because of its superior detectability. Recently, it was reported that the etiology of CMVO is so diverse that its treatment requires an individualized approach [32]. CMR can assess the heart from multiple perspectives, providing both organic and functional regional information. This comprehensive assessment enhances the understanding of the extent and severity of myocardial damage following PCI. Moreover, our proposed non-invasive and accurate detection method of CMVO is expected to be valuable for the following aspects: (1) prognostic prediction: the presence of CMVO has been reported to increase the risk of heart failure, LV remodeling, and sudden cardiac death, allowing early identification of high-risk patients; (2) acute treatment strategies: the presence of CMVO indicates the severity of acute myocardial damage and may require individualized treatment, such as cardioprotective drugs, enhanced rehabilitation programs, and enhanced monitoring of cardiac function; and (3) long-term care planning: the presence of CMVO indicates the severity of myocardial impairment in the chronic phase and may help in the post-PCI recovery period, lifestyle guidance, and coordination of drug therapy. Given these considerations, the detection of CMVO has the potential to be a powerful tool for risk stratification and patient management in clinical practice.

This study had some limitations. First, this was a single-center study with a small sample size, limiting the evaluation of generalization performance. Further studies are needed to validate generalizability in larger populations. Second, the study lacked T1 mapping, which provides valuable insights into myocardial injury and changes in extracellular volume. The absence of T1 mapping, which offers tissue-specific longitudinal relaxation information, may result in an incomplete understanding of CMVO-related myocardial injury. Future large-scale studies that include T1 mapping will help clarify the impact of myocardial injury on CMVO and improve the construction of a model with better generalization performance, thereby strengthening the validity of our findings. Third, this study did not incorporate myocardial infarct size into the model, leaving open the possibility that the parameters might have been confounded by infarct size. Since the primary aim of this study was to evaluate the accuracy of CMVO detection using noninvasive methods, myocardial infarct size, which typically requires the use of CM, was excluded from the analysis. However, given that infarct size is a critical factor associated with the severity of myocardial injury, further research is warranted to investigate its potential confounding effects on the evaluated parameters.

## Conclusions

We assessed the ability of three sets of parameters without CM to detect CMVO in patients with AMI who underwent PCI. Our proposed Top_5 CMR parameters have the potential to diagnose CMVO with high accuracy and without a contrast burden on the kidneys. Furthermore, segmental CMR parameters may identify the location of the CMVO. Non-contrast enhanced multiparametric CMR is expected to be a useful marker for risk stratification, patient management, and treatment decision-making.

## Abbreviations

3D: 3-Dimensional
AMI: Acute myocardial infarction
AUC: Area under curve
CAG: Coronary angiography
CI: Confidence intervals
CM: Contrast media
CMR: Cardiac magnetic resonance
CMVO: Coronary microvascular functional and structural obstruction
CS: Circumferential strain
CVDs: Cardiovascular diseases
DTB: Door to balloon time
EGE: Early gadolinium enhancement
FA: Flip angle
FOV: Field of view
IQR: Interquartile range
LGE: Late gadolinium enhancement
LV: Left ventricular
LVEDVi: Left ventricle endo-diastolic volume index
LVEF: Left ventricle ejection fraction
LVESVi: Left ventricle endo-systolic volume index
OTB: Onset to balloon time
PCI: Percutaneous coronary intervention
ROC: Receiver-operator characteristics
ROI: Region of interest
SA: Short-axis
SENSE: Sensitivity encoding
T1W: T1-weighted
TIMI: Thrombolysis in myocardial infarction
